# An Accord Between Man and Machine: Concordance Between Traditional and Novel Mapping Techniques for Atrioventricular Nodal Reentrant Tachycardia Ablation

**DOI:** 10.7759/cureus.84746

**Published:** 2025-05-24

**Authors:** Phanindra Antharam, Harini Lakshman, Steven Cotteau, Christian Machado

**Affiliations:** 1 Cardiovascular Medicine, Henry Ford Health System, Southfield, USA; 2 Cardiology, Henry Ford Health System, Southfield, USA; 3 Electrophysiology, Henry Ford Health System, Southfield, USA

**Keywords:** 3d-mapping, algorithm, avnrt ablation, cardiac arrhythmias, radio-frequency ablation

## Abstract

Introduction

Catheter ablation has evolved rapidly, starting with conventional anatomic techniques, followed by electrogram mapping, and now isochronal late activation mapping techniques are currently in practice. Success rates of ablation were higher with electrogram mapping compared to conventional anatomic mapping. Conventional techniques performed by an experienced operator have not previously been compared to novel mapping techniques in this cohort.

Methods

A total of 14 consecutive patients underwent Atrioventricular Nodal Reentry Tachycardia (AVNRT) (supraventricular tachycardia with ventriculoatrial (VA) interval <70 ms) ablations, where the operator predicted slow and fast pathway collision points, and a sinus collision mapping was also obtained. Ablation was performed with the operator blinded to mapping. Criteria for successful prediction were an ablation point within 4 mm of machine prediction, with a post-ablation junctional response; slow pathway elimination, confirmed by the absence of an Atrio-His jump with or without an echo beat; and non-inducibility of AVNRT. Other secondary outcomes included age, sex, total radiofrequency (RF) ablation time, number of RF applications, total fluoroscopy time, dose, and other postoperative complications or death.

Results

Operator prediction of sinus collision location coincided with machine prediction in 85.7% of cases. Regarding patient demographics, 57% of the population were female, with a mean age of 60 years. The average distance from operator prediction to machine prediction was 1.75 mm. The percentage of junctional rhythm post-ablation in concordant patients was 83.3%. The mean ablation time was 97 seconds, with seven RF applications on average. Fluoroscopy was used in two patients, with minimal exposure. No post-procedure complications, such as pericardial effusion or atrioventricular (AV) block, were noted.

Conclusion

Conventional techniques were not previously compared with novel mapping techniques. In our retrospective cohort study, there was a concordance of 85.7% between an experienced operator and an algorithm-predicted model. The distance between predicted and actual ablation points was close. Although no concrete predictions can be made given our limited retrospective data, with many limitations, novel mapping techniques are useful tools that currently supplement AVNRT ablations and will likely play a crucial role in the future.

## Introduction

Cardiac electrophysiology, as a field, is advancing at an enormous pace, with new technologies being developed and used for interventions. A glimpse of these advancements may be seen in Atrioventricular Nodal Reentry Tachycardia (AVNRT) ablations, where ablations were initially performed based on anatomical landmarks, but have now evolved to incorporate electroanatomical mapping (EAM) support, greatly enhancing the success rates of these procedures. Although we have previous data showing the feasibility and success rates with the use of EAM in AVNRT ablations, the prediction of ablation sites by EAM - compared to an experienced operator using traditional techniques at an intermediate-volume tertiary care center - has not been previously observed [1]. Given that the use of technology varies by the operator’s training, experience, and expertise, data obtained at a high-volume center may not be reflective of the success rates in centers with lower volumes. Additionally, to our knowledge, the accuracy of EAM in predicting the location or focus of ablation has not been previously reported.

This study was performed with the goal of comparing an experienced operator to an EAM in predicting the focus of ablation and the overlap between the operator and the EAM. We looked into a cohort of patients who underwent AVNRT ablations with EAM of the right atrium, where the operator was blinded to the EAM results and performed the ablation based on a relatively traditional technique. While blinded, the operator predicted the location for ablation, which was later revealed, and the distance between both foci was observed. This data provided insight into the concordance, or agreement, between the operator and the EAM. The operator had 21 years of experience in the field of electrophysiology. The findings of this study may be more pertinent to institutions and communities with access to resources such as EAM; however, there is increasing distribution of these technologies into communities with limited resources over time.

## Materials and methods

The study involved a retrospective review of consecutive cases performed at Ascension Providence Southfield, which is a 628-bed tertiary care center. The informed consent requirement was waived by the IRB, secondary to the retrospective nature of the study. It was conducted in accordance with ethical guidelines and regulations governing research with human subjects. The study was approved by the Institutional Review Board (IRB) of Ascension Providence Southfield (IRB approval number: RMI20230251). AVNRT ablations from June 1, 2022, to June 30, 2023, were reviewed and included in the study, where the operator predicted the ablation site, and a background mapping with the Ensite X system (NavX mode; Abbott Steerable Diagnostic Catheter, Chicago, IL, USA) software was performed. The procedures were reviewed post-procedure, independently by the technician who performed the analysis using the Ensite X software for mapping, and subsequently reviewed for data extraction.

During the study period, 87 AVNRT ablations were done using the guidance of Ensite X software in the facility, of which a total of 15 patients were identified where the operator was blinded to the low voltage bridge/sinus collision point (LVB/SCP) data and reviewed for the study, which was a primary requirement for inclusion. Inclusion into the study required use of the Ensite X system in the background providing EAM, SCP, and LVB mapping data; the operator had to have performed slow pathway ablation using traditional techniques and EAM for navigation but was blinded to the LVB/SCP data. One of the 15 was excluded as they did not have the information to allow for interpretation of concordance.

Data extracted included age, sex, total radiofrequency (RF) ablation time, total fluoroscopy time (if used), intraoperative or post-pericardial effusion, atrioventricular (AV) block, previous ablations, concordance between operator and machine-predicted location, distance between both points, and occurrence of junctional rhythm during RF delivery in the slow pathway zone. No data was collected with regards to follow-up post-procedure, secondary to the cross-sectional nature of the study. 

The data were analyzed by appropriate statistical methods. Percentages were used to compare outcomes such as age and sex distributions, rates of concordance, and presence of junctional rhythm during RF. Averages were used to compare distances between traditional method ablation points and predictions by mapping. Medians were used to show ablation times. Descriptive statistics were reported for other outcomes, such as fluoroscopy use, fluoroscopy dose, and complications including pericardial effusions, AV block, and death. Only descriptive statistics were used, given the nature of the study.

Ensite X mapping

For all AVNRT ablations, a 3D EAM of the right atrium and coronary sinus (CS) was performed using the Ensite X system (NavX mode). Once AVNRT was identified, a voltage and activation timing map was obtained. Atrial voltage points were collected using the 3D geometry and contact mapping of the atrial signals. A steerable diagnostic catheter was primarily used within the triangle of Koch to obtain this data. During the procedure, high voltage and low voltage sliders were typically set at 0.1 mV to 0.5 mV, respectively, to assist with either low- or high-voltage differentiation of atrial tissue [2]. The low-voltage area of interest was classified as 0.5 mV or less by the operator. The low-voltage slider was adjusted to assist with the identification of the low-voltage area. A local activation timing (LAT) map was used to identify the point of the sinus collision, or wave propagation collision point, and a peak-to-peak voltage map was used to assess the LVB that corresponds to the site of the slow pathway zone. The site of sinus collision and LVB overlap is considered the site of the slow pathway, which is the theoretical target for ablation. The mapping procedural steps are done in the background while the operator performs the ablation using traditional methods. The distance between the actual ablation point and the LVB/SCPs was compared subsequently at the end of the procedure.

Traditional/hybrid method

The operator used a combination of EAM and local electrogram guidance for identification of slow conduction pathways; if needed, fluoroscopic guidance was also used. However, EAM from the EnsiteX system was used predominantly, and rarely the fluoroscopic approach. Anatomically, the slow pathway was expected to be located in the septal or postero-septal position, anterior to the roof of the CS and posterior to the HIS bundle tracing past the tricuspid annulus. A quadripolar catheter (Abbott Response JSN 5F, Chicago, IL, USA) was initially used in the high right atrium (HRA), along with a decapolar catheter (Abbott Inquiry Decapolar 5F, Chicago, IL, USA) in the CS, and another quadripolar catheter (Abbott Supreme CRD-Z 5F, Chicago, IL, USA) in the HIS region. The target based on electrogram would include a fractionated atrial signal that is at least, or no more than, one-fourth of the amplitude of the ventricular signal in the same location [2,3].

Steps for ablation

A TactiCath contact force ablation catheter (Abbott TactiCath FJ 8F, Chicago, IL, USA) is maneuvered until a stable, ideal local electrogram signal with an A-to-V ratio of approximately 0.5 is achieved. The area was ablated using an irrigated TactiCath catheter at 30 W, with at least 5 g of force. The flow of irrigation is turned down to 6 mL/min during ablation to ensure a superficial lesion [4]. Energy is delivered for 20 to 30 seconds. Successful ablation was determined by a junctional response lasting at least one minute within the first five seconds of application. If accelerated junctional rhythm was not achieved within the first five seconds, the catheter would be moved anteriorly about 2-3 mm, where a second RF application is delivered at 30 Watts, with achievement of accelerated junctional rhythm, followed by subsequent applications until the same result is achieved.

Concordance definition

Concordance was defined based on a distance of 4 mm between operator-predicted and machine-predicted points, which was measured manually for the purpose of this study. This distance of 4 mm was arbitrary; however, it was based on the premise that an ablation by catheter usually causes damage to tissue about 4 mm circumferentially from the point of contact with cardiac tissue [5]. 

## Results

A total of 14 patients were included in the analysis over the study period. The mean age of the population was 60 years, with a higher percentage of females (57%) and a mean BMI of 29.2. The cohort had comorbidities: six (42.8%) of 14 patients had hypertension, five (35.7%) had hyperlipidemia, one (7.14%) had coronary artery disease, and one (7.14%) had atrial fibrillation. Only one patient in the cohort had congenital heart disease, and the same individual had a history of cardiac surgery. Two (14.2%) of these patients had a prior AVNRT ablation; one of the two had a history of congenital heart disease (Table 1). The primary objective of concordance, as defined previously, was found in 12 (85.7%) of the total patients. This was perceived to be significant; however, the statistical significance of this finding cannot be ascertained given the nature of the study. The percentage of junctional rhythm post-ablation in the total cohort was 71.4% (10 patients). Of the two patients with repeat ablations, one had concordance, but the other did not.

**Table 1:** Demographic Characteristics of Study Participants AVNRT, Atrioventricular Nodal Reentry Tachycardia

Variable	N (%)
Mean age	60 years
Males	6 (43.9%)
Females	8 (57.1%)
BMI (mean)	29.2
Hypertension	6 (42.8%)
Hyperlipidemia	5 (35.7%)
Coronary artery disease	1 (7.14%)
Atrial fibrillation	1 (7.14%)
Congenital heart disease	1 (7.14%)
Previous cardiac surgery	1 (7.14%)
Antiarrhythmic therapy	0
Previous AVNRT ablation	2 (14.2%)

Other parameters that were obtained include a mean ablation time of 97 ms and a median of 5.5 applications per patient. These findings provide insight into the experience and expertise of the operator. Fluoroscopy was used minimally in the cohort, given the 3D map provided by the EAM. It was used in two patients due to technical difficulties with the advancement of equipment, with an average fluoroscopy dose of 6.5 uGym². The average distance between operator prediction and machine prediction was 1.75 mm, with a median of 1.5 mm (Table 2), which also demonstrates the high concordance between the operator's and the mapping algorithm’s predictions. No complications - including intra- or postoperative pericardial effusions, AV block, or death - were noted in the cohort, further reflecting the operator’s experience.

**Table 2:** Procedural Outcomes and Machine-Operator Concordance Results

Variable	N (%)
Concordance operator vs. machine	12 (85.7%)
Junctional rhythm occurrence	10 (71.4%)
Mean ablation time	97 seconds
Median number of applications per patient	5.5
Fluoroscopy assistance	2
Average fluoroscopy exposure	6.5 uGym^2^
Mean distance - operator vs. machine	1.75 mm
Median distance - operator vs. machine	1.5 mm

## Discussion

The AV node is a subendocardial structure measuring about 1 × 3 × 5 mm, located in the infero-posterior right atrium, bordered posteriorly by the CS ostium, superiorly by the tendon of Todaro, and anteriorly by the septal tricuspid valve annulus, referred to as the triangle of Koch [6,7]. AVNRT is a type of supraventricular tachycardia occurring secondary to the presence of a reentry circuit within or adjacent to the AV node. It arises due to the presence of dual or multiple AV nodal pathways, typically referred to as "fast" and "slow" pathways, which differ in conduction velocity and refractory period. The anatomy of the reentrant circuit defines the type of AVNRT: slow-fast AV nodal pathway occurs in about 81.4%, fast-slow in 4.9%, and slow-slow in about 13.7% [8,9]. The reentry occurs because of differences in conduction velocity and refractory period of the pathways [10-12]. The differences in subforms are based on the location of the earliest atrial activation. Based on extensive anatomic and electrophysiologic studies performed previously, the slow pathway was noted to be in the posterior segment of the AV nodal tissue [13,14].

Ablations of the slow pathway for AVNRT termination have evolved over time, with the earliest attempts involving surgical dissection in Koch’s triangle [14-18]. Catheter ablation was first introduced in 1981, and subsequently, success was reported in 1989 with high-energy discharge in the region of the slow pathway [19-21]. Initially, the fast pathway was targeted with RF application superior and posterior to the HIS bundle region. Energy was applied until the occurrence of AV nodal conduction prolongation, with a success rate of 80%-90%; however, the incidence of AV block was as high as 21% of patients [3,22,23]. Slow pathway ablation was subsequently introduced by Jackman et al., with a success rate of 99% and a 0.4% incidence of AV block [24,25]. There were further advancements with the use of electrogram mapping-assisted ablation. One of the studies noted it to be more effective when compared to the anatomical approach, with the anatomic approach being ineffective in approximately 11%-15% of patients [1,3,26]. Propagation mapping for use in AVNRT ablations was described by Bailin et al. in 2011, and subsequently by other studies, as a viable adjunct to anatomical and electrophysiological criteria for catheter ablations [27,28]. 

In our study, we aimed to compare an experienced operator to the prediction by the mapping algorithm for accurate identification of the slow pathway, which is represented by the overlapping sites of sinus collision and LVB. The variable used to measure this was the amount of concordance, measured by the distance between the operator's predicted ablation site and the machine's prediction of the slow pathway. The distance for concordance in our study was arbitrary but based on the expected range of tissue destruction, which is about 4 mm secondary to ablation. Based on our definition, we noted that about 86% (12/14) of patients had concordance between operator and machine prediction. The results provide insight into the accuracy of current technology in prediction compared to techniques used by an experienced operator, which are based on anatomical landmarks and intracardiac electrical potentials. The operator in the cohort has also used the mapping system instead of fluoroscopy to help obtain anatomical landmarks in conjunction with electrical potentials, helping reduce the use of fluoroscopy and thus the amount of radiation exposure to the patient, as evidenced by the very low frequency of fluoroscopy use - about two patients in the total cohort - and the low average radiation dose of 6.5 uGym². Fluoroscopy was used due to difficulty with advancement of equipment on retrospective review with the operator in both cases.

Mean age and sex in the study population were comparable to those in other studies. Mean RF ablation time was 97.4 seconds post-ablation, which was significantly less when compared to a large study (reported about 208 to 224 seconds) and reflects the operator's expertise and experience with the traditional method of AVNRT ablation [29].

Although we tried to compare a traditional method to the use of mapping software for the prediction of the slow pathway, the operator in our case series opted to use the EAM produced by the Ensite X system to minimize the use of fluoroscopy, which is the basis of the traditional method for AVNRT ablations. The use of the mapping system does lower fluoroscopy use, as shown in our cohort, where only two patients needed fluoroscopy and a very low dose of radiation when it was used. This makes the approach less traditional and more of a hybrid one; however, it demonstrates the capabilities of the Ensite X system to provide an accurate anatomical map without fluoroscopy. The technique aligns more with traditional methods as it uses anatomical landmarks and intracardiac electrograms to perform the ablation.

There are significant limitations to using the data from our study, as it is a single-center, retrospective cohort study with a limited number of patients and procedures performed by a single operator. The use of EAM instead of fluoroscopy for anatomical landmarks also introduces significant limitations, as we are not comparing a traditional technique - albeit a hybrid one - with the prediction algorithm. The determination of the slow pathway by the EAM was done in the background; however, there was no standardization for this process, and there is potential for unblinding of the data to the operator, which could potentially increase the percentage of overlap and thus falsely increase concordance reported in the study. The concordance was measured manually by a technician, which could have confounded the results and falsely increased the concordance due to subconscious bias to increase overlap between EAM and operator. The concordance, though defined by a range of tissue destruction by an ablation catheter, is arbitrary and not a standardized measure. There is also potential for selection bias, as only the patients meeting the said inclusion criteria were included; however, the cases may have been selected by the operator subconsciously. 

AVNRT ablations have come a long way - from causing significant complications and acceptable success rates to almost no complications with near 100% success rates [29]. All procedures come with learning curves and variable success rates that differ from one operator to another. Technological advancements have increasingly assisted medical procedures, thus decreasing procedural time and improving procedural safety over the years. The EAM provided by the mapping algorithms during AVNRT ablations in our institution was similar enough to anatomical landmarks obtained by traditional fluoroscopy to be used by the operator in the study. Despite its limitations, our study provides data showing that the algorithms identifying the slow pathway location are comparable to predictions by an experienced operator. Based on our experience and data, we expect these algorithms and novel mapping techniques to assist and improve success rates through improved accuracy and decreased complications for any operator who chooses to use them - especially in relatively lower-volume institutions. We believe they will help reduce inter-operator variability and increase procedural success, even more so when used on a global scale, where access to this technology is feasible. We hope the adoption of these techniques will help provide uniform, standardized, and personalized medicine/interventions to all patient populations. With the advent of machine learning or artificial intelligence (AI) programs, we expect these assistance programs to grow exponentially in the coming years. Currently, mapping is performed by a technician in the procedure room, but this may change with advancements in AI systems taking the place of the technician. This may also result in better prediction algorithms and thus higher success rates for these procedures.

## Conclusions

Use of 3D EAM as a way of navigation and delivering therapy is increasingly used in the field of electrophysiology and has been empowering physicians who perform ablation procedures to provide improved success rates with lower complications for their patients. Although these systems have proven their benefit, a pragmatic, real-world comparison to an operator outside of a high-volume center was not performed previously. Based on our results, we believe that mapping software in AVNRT ablations can be a useful tool to help provide navigation and ablation site input comparable to an experienced operator. We predict it will likely play a crucial role in the future with advancements in technology.

## References

[1] Kalbfleisch SJ, Strickberger SA, Williamson B (1994). Randomized comparison of anatomic and electrogram mapping approaches to ablation of the slow pathway of atrioventricular node reentrant tachycardia. J Am Coll Cardiol.

[2] Devecchi C, Matta M, Magnano M (2024). Voltage and propagation mapping: new tools to improve successful ablation of atrioventricular nodal reentry tachycardia. J Cardiovasc Electrophysiol.

[3] Jazayeri MR, Hempe SL, Sra JS (1992). Selective transcatheter ablation of the fast and slow pathways using radiofrequency energy in patients with atrioventricular nodal reentrant tachycardia. Circulation.

[4] Petersen HH, Chen X, Pietersen A, Svendsen JH, Haunsø S (2000). Tissue temperatures and lesion size during irrigated tip catheter radiofrequency ablation: an in vitro comparison of temperature-controlled irrigated tip ablation, power-controlled irrigated tip ablation, and standard temperature-controlled ablation. Pacing Clin Electrophysiol.

[5] Verma A, Maffre J, Sharma T, Farshchi-Heydari S (2025). Effect of sequential, colocalized radiofrequency and pulsed field ablation on cardiac lesion size and histology. Circ Arrhythm Electrophysiol.

[6] Inoue S, Becker AE (1998). Koch's triangle sized up: anatomical landmarks in perspective of catheter ablation procedures. Pacing Clin Electrophysiol.

[7] Sumitomo N, Tateno S, Nakamura Y (2007). Clinical importance of Koch's triangle size in children: a study using 3-dimensional electroanatomical mapping. Circ J.

[8] Marzlin KM (2017). Atrioventricular nodal reentrant tachycardia. AACN Adv Crit Care.

[9] Heidbüchel H, Jackman WM (2004). Characterization of subforms of AV nodal reentrant tachycardia. Europace.

[10] Goldreyer BN, Bigger JT Jr (1971). Site of reentry in paroxysmal supraventricular tachycardia in man. Circulation.

[11] Goldreyer BN, Damato AN (1971). The essential role of atrioventricular conduction delay in the initiation of paroxysmal supraventricular tachycardia. Circulation.

[12] Denes P, Wu D, Dhingra RC, Chuquimia R, Rosen KM (1973). Demonstration of dual A-V nodal pathways in patients with paroxysmal supraventricular tachycardia. Circulation.

[13] McGuire MA, Robotin M, Yip AS (1994). Electrophysiologic and histologic effects of dissection of the connections between the atrium and posterior part of the atrioventricular node. J Am Coll Cardiol.

[14] Medkour D, Becker AE, Khalife K, Billette J (1998). Anatomic and functional characteristics of a slow posterior AV nodal pathway: role in dual-pathway physiology and reentry. Circulation.

[15] Ross DL, Johnson DC, Denniss AR, Cooper MJ, Richards DA, Uther JB (1985). Curative surgery for atrioventricular junctional (“AV nodal”) reentrant tachycardia. J Am Coll Cardiol.

[16] Cox JL, Holman WL, Cain ME (1987). Cryosurgical treatment of atrioventricular node reentrant tachycardia. Circulation.

[17] Guiraudon GM, Klein GJ, van Hemel N, Guiraudon CM, Yee R, Vermeulen FE (1990). Anatomically guided surgery to the AV node. AV nodal skeletonization: experience in 46 patients with AV nodal reentrant tachycardia. Eur J Cardiothorac Surg.

[18] Ruder MA, Mead RH, Smith NA, Gaudiani VA, Winkle RA (1991). Comparison of pre- and postoperative conduction patterns in patients surgically cured of atrioventricular node reentrant tachycardia. J Am Coll Cardiol.

[19] Scheinman MM, Morady F, Hess DS, Gonzalez R (1982). Catheter-induced ablation of the atrioventricular junction to control refractory supraventricular arrhythmias. JAMA.

[20] Haissaguerre M, Warin JF, Lemetayer P, Saoudi N, Guillem JP, Blanchot P (1989). Closed-chest ablation of retrograde conduction in patients with atrioventricular nodal reentrant tachycardia. N Engl J Med.

[21] Epstein LM, Scheinman MM, Langberg JJ, Chilson D, Goldberg HR, Griffin JC (1989). Percutaneous catheter modification of the atrioventricular node. A potential cure for atrioventricular nodal reentrant tachycardia. Circulation.

[22] Lee MA, Morady F, Kadish A (1991). Catheter modification of the atrioventricular junction with radiofrequency energy for control of atrioventricular nodal reentry tachycardia. Circulation.

[23] Langberg JJ, Leon A, Borganelli M, Kalbfleisch SJ, El-Atassi R, Calkins H, Morady F (1993). A randomized, prospective comparison of anterior and posterior approaches to radiofrequency catheter ablation of atrioventricular nodal reentry tachycardia. Circulation.

[24] Jackman WM, Beckman KJ, McClelland JH (1992). Treatment of supraventricular tachycardia due to atrioventricular nodal reentry by radiofrequency catheter ablation of slow-pathway conduction. N Engl J Med.

[25] Morady F (2004). Catheter ablation of supraventricular arrhythmias: state of the art. J Cardiovasc Electrophysiol.

[26] Kay GN, Epstein AE, Dailey SM, Plumb VJ (1992). Selective radiofrequency ablation of the slow pathway for the treatment of atrioventricular nodal reentrant tachycardia. Evidence for involvement of perinodal myocardium within the reentrant circuit. Circulation.

[27] Bailin SJ, Korthas MA, Weers NJ, Hoffman CJ (2011). Direct visualization of the slow pathway using voltage gradient mapping: a novel approach for successful ablation of atrioventricular nodal reentry tachycardia. Europace.

[28] Van Aartsen A, Law IH, Maldonado JR, Von Bergen NH (2017). Propagation mapping wave collision correlates to the site of successful ablation during voltage mapping in atrioventricular nodal reentry tachycardia. J Innov Card Rhythm Manag.

[29] Chrispin J, Misra S, Marine JE (2018). Current management and clinical outcomes for catheter ablation of atrioventricular nodal re-entrant tachycardia. Europace.

